# Three Landmark Optimization Strategies for Mobile Robot Visual Homing

**DOI:** 10.3390/s18103180

**Published:** 2018-09-20

**Authors:** Xun Ji, Qidan Zhu, Junda Ma, Peng Lu, Tianhao Yan

**Affiliations:** College of Automation, Harbin Engineering University, Harbin 150001, China; zhuqidan@hrbeu.edu.cn (Q.Z.); aa285136149@gmail.com (J.M.); lupengpeng@hrbeu.edu.cn (P.L.); 1825109095@hrbeu.edu.cn (T.Y.)

**Keywords:** mobile robot navigation, visual homing, panoramic vision, scale-invariant feature transform

## Abstract

Visual homing is an attractive autonomous mobile robot navigation technique, which only uses vision sensors to guide the robot to the specified target location. Landmark is the only input form of the visual homing approaches, which is usually represented by scale-invariant features. However, the landmark distribution has a great impact on the homing performance of the robot, as irregularly distributed landmarks will significantly reduce the navigation precision. In this paper, we propose three strategies to solve this problem. We use scale-invariant feature transform (SIFT) features as natural landmarks, and the proposed strategies can optimize the landmark distribution without over-eliminating landmarks or increasing calculation amount. Experiments on both panoramic image databases and a real mobile robot have verified the effectiveness and feasibility of the proposed strategies.

## 1. Introduction

In robotic community, the topic of autonomous mobile robot navigation has been widely discussed for decades, and various navigation schemes based on different types of sensors have been proven to be effective and employed in practical applications [1,2,3,4,5]. Among the numerous vision-based autonomous navigation solutions, visual homing has drawn extensive attention for its simple model and high navigation accuracy. Using visual homing approaches, the robot can be guided to the specified destination only by comparing its currently captured panoramic image with the pre-stored target image [6,7,8,9].

Visual homing is inspired by biological navigation, which can be considered as a kind of qualitative autonomous navigation technique [10,11]. In contrast to the most popular visual SLAM technology [12,13,14,15], visual homing does not require any localization or mapping. Instead, it only needs to continuously calculate the direction vector, known as the homing vector, pointing from the current location to the destination. In summary, the robot can achieve the homing task by solely repeating the following three steps: (1) relating the current panoramic image to the target panoramic image; (2) computing the homing vector; and (3) moving based on the calculated homing vector [16,17,18].

Visual homing requires only one type of input called landmark. In the early stage, researchers often adopted distinctive objects (such as black or brightly colored cylinders) in the environment as artificial landmarks [19,20]. However, since the identification of such landmarks must be manually interfered, this would inevitably limit the application scope, and natural landmarks thus gradually became a better choice. Some homing approaches adopted pixels at fixed locations in the panoramic images as natural landmarks [21,22]. This solution proved to be feasible, but a problem was unsolved that the homing performance would drop sharply when illumination changed during the movement of the mobile robot. With the development of computer vision technology, image feature extraction algorithms were proposed, such as SIFT [23] and speed-up robust features (SURF) [24]. The SIFT and SURF features have two outstanding advantages. First, these features are highly distinctive and can be correctly matched with high probability. Second, these features can exhibit excellent invariance to affine distortion, addition of noise and change in illumination [25]. Currently, numerous visual homing approaches use SIFT or SURF features as natural landmarks to complete navigation tasks in practical applications [26,27,28].

Aiming at guiding the robot to the specific destination accurately, different types of visual homing algorithms have been proposed. Among them, warping and average landmark vector (ALV) are the two most representative homing approaches. Both warping and ALV have been verified to exhibit good homing performance and robustness. These two homing approaches are widely used in practical applications, and many advanced algorithms based on them have been presented.

The warping methods distort either of the panoramic images (usually the target image) based on the parameters describing the distance, direction and rotation of the mobile robot’s movement. Multiple parameter combinations produce different forms of the distorted images, and the warping methods will compare all the distorted images with the current image. The optimal parameters will be determined when the distorted image best fits the current image, and in what follows the homing vector can be generated [21]. Based on the original warping method, many advanced warping algorithms have been proposed to further optimize the homing performance. Möller et al. proposed the 2D-warping and min-warping methods, and these two advanced algorithms could achieve the variation of the alignment angle estimation in the environment instead of the external compass in the original 1D-warping [29]. Besides, a SIMD implementation was also adopted based on min-warping so that the tolerance and efficiency can be further enhanced [30,31]. Zhu et al. used SIFT features such as natural landmarks instead of fixed pixels in the image so that the robustness to the illumination can be improved [27]. Recently, several actual experiments were performed to compare these advanced warping methods with other homing approaches in many aspects, such as illumination tolerance and computation speed [32,33,34].

ALV is considered as one of the simplest homing approaches in concept, and it is often used as a benchmark in the field of visual homing. ALV is defined as an average bearing of all the extracted landmarks with respect to a certain location, and the homing vector can be calculated by the difference between the ALV at the current location and the ALV at the target location [20,26]. Thus far, ALV has also been enriched to further optimize the homing performance. Zhu et al. presented an optimal ALV model based on sparse landmarks [35]. Yu et al. analyzed the effect of landmark vectors and proposed an improved ALV algorithm, called Distance-Estimated Landmark Vector (DELV) [36]. Lee et al. adopted the omnidirectional depth information to further enrich DELV [37]. All these advanced homing algorithms have been verified to be effective and feasible, and, under the guidance of these algorithms, mobile robot can move towards the target location with a better trajectory.

As a local navigation strategy, although visual homing exhibits high navigation accuracy in a small-scale environment, it is constrained by the landmark distribution. Most of the visual homing approaches should satisfy as much as possible the equal distance assumption, which describes an ideal landmark distribution state. Due to the lack of depth sensors for traditional visual homing approaches, distance information between key locations and landmarks is difficult to obtain, so the equal distance assumption provides constraint that the landmarks need to be uniformly located the same distance from the target location [28,29]. Although this assumption has been verified to be correct [36], it is always violated in practical applications, and irregularly distributed landmarks will have a negative impact on the performance for visual homing.

The landmark distribution is one of the key factors affecting the homing performance. However, research on the optimization of landmark distribution is very limited. Zhu et al. presented two solutions: The first solution is to establish a task-dependent optimization procedure to distinguish the most relevant landmarks, including the spatial distribution, feature selection and updating [38,39]. The second solution is to optimize the landmark distribution by removing some of the landmarks and preserving only the landmarks that are uniformly distributed [35]. In addition, since the DELV algorithm adopts the depth sensor, it is considered to be able to overcome the constraints of the equal distance assumption so that the possible effects can be reduced [37]. The above three methods can effectively improve the homing accuracy of the robot, but these methods still have some limitations. The first two methods achieve distribution optimization at the expense of discarding landmarks; if the current location is far from the target location, the number of landmarks that can be used as input is too small, thus the probability of potential errors may be increased. Besides, although DELV can exhibit good homing performance, the use of depth sensors will inevitably increase computational complexity and experimental costs.

To avoid the above problems, this paper presents three landmark optimization strategies for mobile robot visual homing. We adopt SIFT features as natural landmarks, and the proposed strategies have two purposes. The first purpose is to eliminate mismatching landmarks (Strategy 1). The second purpose is to optimize the landmark distribution by the idea of weight assignment (Strategies 2 and 3). The proposed strategies evaluate the value of each landmark, and, for the landmarks that are judged to be of low quality, the strategies will assign them lower weights, weakening their contribution to the homing approaches. These strategies do not require any excessive landmark elimination or external sensors, and the effect of the optimal landmark distribution can be achieved by setting a reasonable weight for each landmark. In this paper, we combine our proposed strategies with the ALV model to test their effectiveness.

The paper is summarized as the following sections: In Section 2, we introduce the ALV algorithm in detail. We then present the three landmark optimization strategies in Section 3. In Section 4, we perform a series of experiments on both image databases and a real mobile robot along with the related analysis. In Section 5, we discuss the homing performance of the algorithms. In Section 6, we draw conclusions and point out the future work.

## 2. Average Landmark Vector

**Figure 1:**
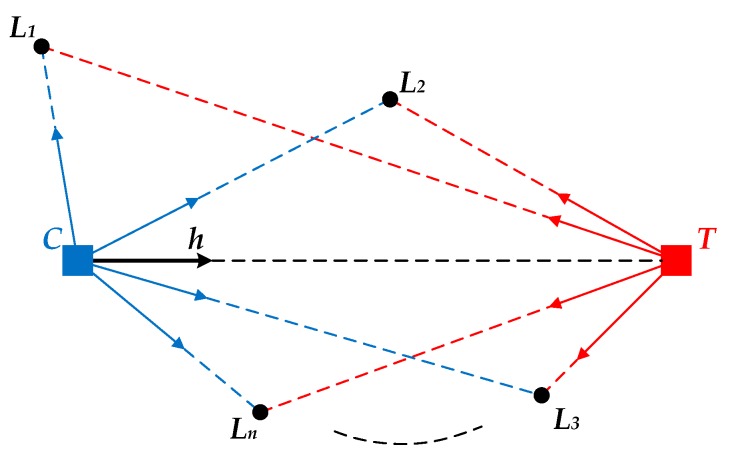
The ALV model. The blue square represents the current location. The red square represents the target location. The black circles represent the extracted landmarks. The blue and red arrows stand for the corresponding landmark vectors. The desired homing vector is denoted as the black arrow.

The ALV model is described in Figure 1. *C* is the current location of the robot. *T* is the target location. *L_i_* is the *i*th landmark, where *i* = 1, 2, …, *n*. ***h*** is the desired homing vector pointing from *C* towards *T*.

For the current image captured at *C*, according to the imaging principle of panoramic vision, it can be indicated that the projection point of *C* will be in the center of the panoramic image. We define *C* as the origin and create the image coordinate system. Assuming that the image coordinate of *L_i_* is $P_{i}^{C}=(x_{i},y_{i})$, the corresponding landmark vector of *L_i_* can thus be calculated by:

(1) $$\vec{CL_{i}}=\frac{P_{i}^{C}-P_{0}^{C}}{\left\|P_{i}^{C}-P_{0}^{C}\right\|}\;$$

where $P_{0}^{C}=(0,0)$ is the image coordinate of *C*. For all the landmarks observed at *C*, the average landmark vector can be computed as follows:

(2) $$\vec{ALV_{C}}=\frac{1}{n}\sum_{i=1}^{n}\vec{CL_{i}}\;$$

Similarly, for the target image captured at *T*, the landmark vector of *L_i_* can be calculated by:

(3) $$\vec{TL_{i}}=\frac{P_{i}^{T}-P_{0}^{T}}{\left\|P_{i}^{T}-P_{0}^{T}\right\|}\;$$

where $P_{i}^{T}$ is the coordinate of *L_i_* in the target image, and $P_{0}^{T}=(0,0)$ is the coordinate of *T*. For all the landmarks observed at *T*, the average vector can be computed as follows:

(4) $$\vec{ALV_{T}}=\frac{1}{n}\sum_{i=1}^{n}\vec{TL_{i}}\;$$

Finally, the unit homing vector can be obtained by the subtraction of the average landmark vector at *C* and *T*:

(5) $$h=\frac{\vec{ALV_{C}}-\vec{ALV_{T}}}{\left\|\vec{ALV_{C}}-\vec{ALV_{T}}\right\|}\;$$

ALV is a conceptually simple visual homing approach with an easy-to-understand model, and the desired homing vector can be generated only by vector calculation. However, ALV is greatly affected by the landmark distribution. Since all the landmark vectors are set to unit vectors in the original ALV algorithm, the calculated homing vector will be close to the ideal homing vector only when the distance from the target location to each landmark is approximately equal.

**Figure 2:**
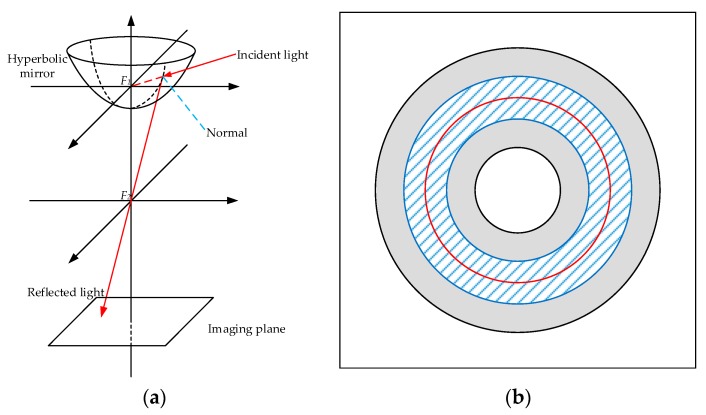
Simplified model of panoramic vision system and imaging plane: (**a**) simplified model of panoramic vision system including hyperbolic mirror and image plane; and (**b**) imaging plane. The red circle is horizon circle. In the SL-ALV algorithm, the landmarks located in the blue shaded area are selected as the input while the landmarks located in the grey area are removed. The white parts denote the invalid areas in the panoramic image.

To solve the landmark distribution problem of ALV, an improved algorithm was proposed based on sparse landmarks, abbreviated as SL-ALV in this paper [35]. We will introduce SL-ALV in detail because we use it as a comparative method with our proposed strategies. SL-ALV optimizes the landmark distribution inspired by the imaging principle of panoramic vision system, which is shown in Figure 2. The panoramic vision system contains a hyperbolic mirror, which reflects all the incident lights onto the imaging plane. *F*_1_ and *F*_2_ are two foci of the system. According to the imaging principle, all extensions of the incident lights will converge on *F*_1_, and all reflected lights will pass through *F*_2_. A crucial phenomenon can be stated that, for landmarks with the same height as *F*_1_ in the panoramic vision system, since the angles between all the corresponding incident lights and the hyperbolic mirror are constant, the angles between the reflected lights and the imaging plane will also remain the same. Hence, the projection pixels of these landmarks must be on a fixed circle of the image, called horizon circle. Wherever the system moves, these projection pixels will not leave the horizon circle.

Inspired by the above phenomenon, SL-ALV considers the projection pixels (i.e., landmarks) located near the horizon circle to be relatively more valuable. On the one hand, the horizon circle can provide a reference for the optimal landmark distribution, and the projection pixels near the horizon circle are substantially the same image distance from the center of the image. On the other hand, when the panoramic vision system moves, the projection pixels near the horizon circle will not have a large shift in image position so that the stability of the landmark distribution can be better ensured. In the SL-ALV algorithm, the horizon circle is properly expanded to form a ring area (the width of the ring area is usually set to half the width of the valid area, shown as Figure 2b), and all the landmarks located outside the ring area will be discarded. Therefore, the remaining landmarks can better satisfy the equal distance assumption, and SL-ALV can thus improve the homing accuracy compared to the original ALV algorithm. However, SL-ALV inevitably reduces the total number of landmarks, which may lead to a potential problem that the small sample sizes will cause unpredictable errors. This problem is particularly evident when the current location is far from the target location, because the similarity of the two panoramic images is low in this case, and this will result in a very limited number of the extracted landmarks.

## 3. Landmark Optimization Strategies

In this section, we introduce the three landmark optimization strategies in detail, including mismatching landmark elimination, landmark contribution evaluation and multi-level matching. We use SIFT features as natural landmarks, and the optimized landmarks have higher accuracy and more reasonable distribution.

### 3.1. Strategy 1: Mismatching Landmark Elimination

Strategy 1 is presented to improve the overall accuracy of the extracted landmarks. Based on the SIFT scale principle and panoramic image principle, Strategy 1 can effectively detect and eliminate the potential mismatching landmarks to further optimize the performance of the visual homing approaches.

#### 3.1.1. SIFT Scale Principle

As mentioned in [28], a noteworthy conclusion is drawn that the SIFT scale value is negatively related to the spatial distance from the landmark to the viewer. That is to say, if the viewer is closer to a certain landmark, the SIFT feature, which refers to this landmark, will have a larger scale value. This is because scale indicates the degree of Gaussian blurring required to reveal the distinctive characteristic of the feature. When the landmark is approached, its corresponding SIFT feature need to be detected with a higher degree of Gaussian blurring. Therefore, for the same SIFT feature matching pair, we can qualitatively estimate the distance from the landmark to the two capture positions by comparing the scale values of the two SIFT features.

We use Lowe’s SIFT implementation (available from: http://www.cs.ubc.ca/~lowe/keypoints/) and the *i*th SIFT feature can be described as follows:

(6) $$f_{i}=(f_{i}^{x},f_{i}^{y},f_{i}^{o},f_{i}^{\sigma },f_{i}^{d})\;$$

where $f_{i}^{x}$ and $f_{i}^{y}$ are the image coordinates of $f_{i}$; $f_{i}^{o}$ is the orientation; $f_{i}^{\sigma }$ is the scale; and $f_{i}^{d}$ is the descriptor.

**Figure 3:**
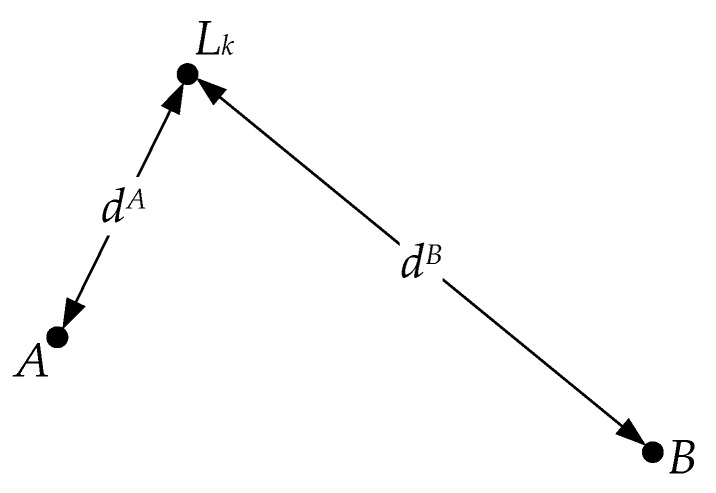
Key locations in the simulated scene. *A* and *B* represent the two capture positions of the viewer, *L_k_* is the *k*th landmark, and *d^A^* and *d^B^* separately denote the distance from *L_k_* to *A* and *B*.

Figure 3 shows the key locations in the simulated scene. Assuming that the viewer captures the panoramic image *I_A_* and *I_B_* at positions *A* and *B*, respectively, when the two images are matched, the *k*th SIFT feature matching pair can be described as follows:

(7) $$p_{k}=(f_{i},f_{j})\;$$

where *f_i_* is the *i*th SIFT features in *I_A_*, and *f_j_* is the *j*th SIFT feature in *I_B_*.

We define Δσ as the difference between the scale values of *f_i_* and *f_j_*:

(8) $$\Delta \sigma =f_{i}^{\sigma }-f_{j}^{\sigma }\;$$

Therefore, *d^A^* and *d^B^* can be qualitatively compared based on Δσ:

(9) $$\left\{ \begin{array}{l} d^{A}>d^{B},\;\mathrm{if}\;\Delta \sigma <0\\ d^{A}<d^{B},\;\mathrm{if}\;\Delta \sigma >0 \end{array}\right.\;$$

#### 3.1.2. Panoramic Imaging Principle

**Figure 4:**
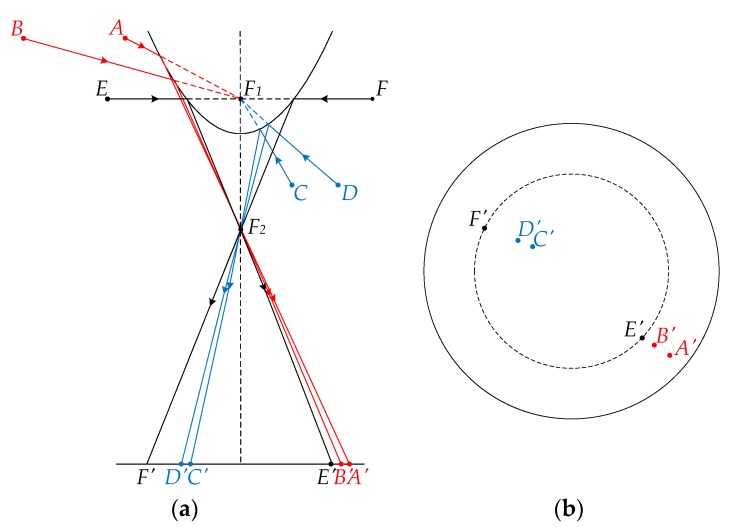
Schematic diagram of panoramic imaging principle. (**a**) Panoramic vision system: *F*_1_ and *F*_2_ are the foci of the panoramic vision system. *A*–*F* are six landmarks in the real scene. The vertical heights of *A* and *B*, *C* and *D*, and *E* and *F* are, respectively, the same, where *A* and *B* are above *F*_1_, *C* and *D* are below *F*_1_, and *E* and *F* are the same height as *F*_1_. *A*’–*F*’ are the corresponding projection points on the imaging plane. (**b**) Top view of the imaging plane: The dotted circle is denoted as the horizon circle.

Generally, almost all visual homing approaches select panoramic images as input because a panoramic vision system can capture the entire 360-degree information in azimuthal direction and close to 180-degree information in unilateral vertical direction [40]. Compared to other types of images, panoramic images always contain more environmental information in space. Figure 4 shows the schematic diagram of panoramic imaging principle, which can be summarized as follows:

(1) If the landmarks with the same vertical height are located above *F*_1_, their projection points will always lie outside the horizon circle, shown as (*A*, *A*’) and (*B*, *B*’). The distance between the projection point and the image center will decrease as the panoramic vision system moves away from the landmark.
(2) If the landmarks with the same vertical height are located below *F*_1_, their projection points will always lie inside the horizon circle, shown as (*C*, *C*’) and (*D*, *D*’). The distance between the projection point and the image center will decrease as the panoramic vision system moves towards the landmark.
(3) If the landmarks are located the same height as *F*_1_, their projection points will always be on the horizon circle, shown as (*E*, *E*’) and (*F*, *F*’)

In conclusion, based on the panoramic imaging principle, we can take advantage of the pixel positions in the panoramic images to qualitatively determine the distance relation from the system to the two capture positions. Assuming that the actual distances from the system to the six landmarks mentioned in Figure 4 are *d^A^*, *d^B^*, *d^C^*, *d^D^*, *d^E^* and *d^F^*, the corresponding pixel distances from the image center to the projection points are *d^A^*^’^, *d^B^*^’^, *d^C^*^’^, *d^D^*^’^, *d^E^*^’^ and *d^F^*^’^. The panoramic principle can thus be formulated as follows:

(10) $$\left\{ \begin{array}{l} d^{A}<d^{B},\;\mathrm{if}\;d^{A’}>d^{B’}>r\\ d^{C}<d^{D},\;\mathrm{if}\;d^{C’}<d^{D’}<r\\ d^{E}=d^{F},\;\mathrm{if}\;d^{E’}=d^{F’}=r \end{array}\right.\;$$

#### 3.1.3. Mismatching Landmark Elimination

Inspired by the above analysis, two principles can be provided to obtain the relative distance relation between the selected landmark and the capture positions, including SIFT scale principle and panoramic imaging principle. Therefore, for each SIFT feature matching pair, if the conclusions drawn by the above two principles are conflicting, it can be inferred that at least one conclusion is mistaken. In this case, Strategy 1 considers that the currently verified SIFT feature matching pair is not reliable, which needs to be discarded. The pseudo code of Strategy 1 is shown in Algorithm 1.

**Algorithm 1.** Pseudo code of Strategy 1.1:  Capture the panoramic images at two different positions.2:  Execute the SIFT detection and matching algorithm 3:  **for** (each SIFT feature matching pair) **do** 4:   Determine the conclusion from SIFT scale principle based on Equation (9)5:   Determine the conclusion form panoramic imaging principle based on Equation (10) 6:   Verify whether the above two conclusions are conflicting 7:   **if** TRUE **then** 8:    Discard the currently verified SIFT feature matching pair 9:   **else** 10:     Reserve the currently verified SIFT feature matching pair 11:    **end if** 12:    **end for**

Strategy 1 is theoretically correct and feasible, but the two images are inevitably affected by some uncertainties in practice applications, such as image resolution, ground flatness, human error, etc. If the scale difference of the two SIFT features in the same matching pair is too approximated, it will be unreliable by using this strategy to determine whether such type of matches is mismatched. Hence, a scale difference threshold $\sigma_{T}$ can be set, and Strategy 1 will only verify the SIFT matches with $\Delta \sigma >\sigma_{T}$. Based on our experimental results, the optimal performance can be obtained when we set $\sigma_{T}=0.5$. However, this value is not constant; $\sigma_{T}$ can be modified appropriately based on the actual situation to exhibit the optimal performance of Strategy 1.

### 3.2. Strategy 2: Landmark Contribution Evaluation

Strategy 2 is applied to optimize the landmark distribution for visual homing. Compared to the traditional optimization algorithm, Strategy 2 does not directly discard the landmarks, but evaluates their contribution to the final result. For the landmarks with low contribution, Strategy 2 will accordingly reduce their weights, thereby optimizing the actual landmark distribution while preserving the existing landmarks.

**Figure 5:**
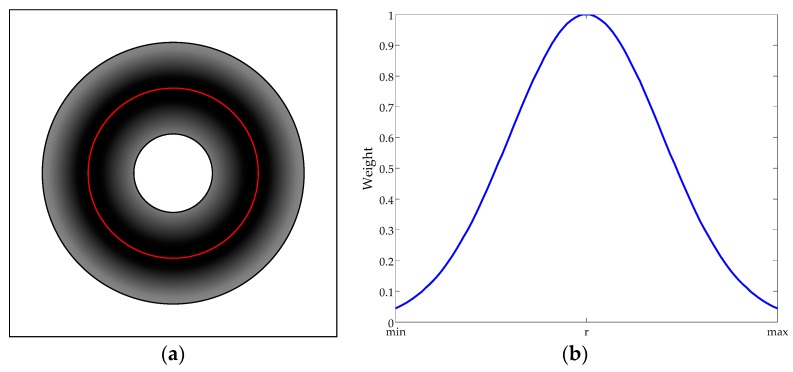
The model of Strategy 2 and weight assignment: (**a**) the model of Strategy 2, where the horizon circle is marked as the red part in the figure; and (**b**) weight assignment.

Strategy 2 adopts the similar idea to SL-ALV. Based on the imaging principle of the panoramic images introduced in Section 2, it can be concluded that the landmarks detected near the horizon circle are more accurate and valuable than those far from the horizon circle. Therefore, Strategy 2 is designed to assign a specific weight to each landmark based on its projected position in the panoramic image. We employ Gaussian function to evaluate the contribution of the landmarks, and the weight of the *i*th landmark *w_i_* can be calculated as follows:

(11) $$w_{i}=\frac{1}{\sigma_{G}\sqrt{2\pi }}\exp(-\frac{{\left(d_{i}-r\right)}^{2}}{2{\sigma_{G}}^{2}})\;$$

where $\sigma_{G}$ is the standard deviation; *d_i_* is the image distance between the *i*th landmark and the image center; and *r* is the radius of the horizon circle. In this paper, we set $\sigma_{G}=2.0$ according to our own experimental conditions. Similar to the setting of $\sigma_{T}$ in Strategy 1, the value of $\sigma_{G}$ is also optional, and researchers can modify this value based on the actual situation to exhibit the optimal performance of Strategy 2. Figure 5 shows the model of Strategy 2. Landmarks in the darker color area are assigned higher weights, and this type of landmarks will play a more important role in the subsequent homing calculations.

Strategy 2 effectively evaluates the reliability and stability of the landmarks without discarding landmarks, thus solving the potential problems that may arise due to the excessive landmark elimination. Based on Strategy 2, high-quality landmarks will play a key role in the homing approaches, while the effect of low-quality landmarks will be reduced.

### 3.3. Strategy 3: Multi-Level Matching

Strategy 3 is presented according to the key point matching criterion in the SIFT algorithm. By setting multi-level distance ratio thresholds, SIFT features can be extracted in stages, and the features are assigned corresponding weights in different intervals. Finally, the contribution of the features determined to be less accurate is weakened.

#### 3.3.1. Nearest-Neighbor Distance Ratio

Nearest-neighbor distance ratio (NNDR) is a type of key point matching metric for SIFT algorithm. Since the SIFT matches determined by NNDR have extremely high matching accuracy, NNDR has become the most commonly used key point matching criterion in the fields of object recognition and image registration.

When a SIFT feature is detected, the corresponding descriptors are obtained based on the local image gradients measured at the selected scale in the region around the feature. Each descriptor contains 128-dimensional feature vectors, which describe significant levels of local shape distortion and change in illumination [23]. NNDR is applied to find the best candidate match between two images: If the nearest neighbor is defined as the feature with minimum Euclidean distance for the descriptor vector, a possible match can be declared when the distance ratio of the nearest neighbor to the second-nearest neighbor is less than a certain threshold. According to Lowe et al. [23], when the distance ratio threshold is set to 0.8, most of the false matches can be eliminated and only a small number of correct matches are discarded at the same time. As the threshold decreases, the total number of matches that can be obtained will decrease, but the proportion of correct matches will increase.

In this section, we propose an extended NNDR matching criterion, called multi-level matching. The proposed criterion can be specifically used in the field of visual homing. By setting multi-level distance ratio thresholds, the matches declared in different intervals will be given corresponding weights, thus further optimizing the accuracy of the landmarks.

#### 3.3.2. Multi-Level Matching

Strategy 3 selects five distance ratio thresholds to declare matches in stages. Since the number of matches that can be obtained is limited when the threshold is less than 0.4, the candidate thresholds we select are 0.4, 0.5, 0.6, 0.7 and 0.8 in order. The reason we set the thresholds in this way is mainly to provide an easy-to-operate scheme. To avoid excessive computation complexity while ensuring optimization effects, we consider that dividing the intervals evenly is a straightforward and effective solution.

The core idea of multi-level matching can be described as follows: When the first threshold 0.4 is set, the obtained matches are considered to be almost error-free, so the landmarks represented by these matches are assigned a weight of 1. When the second threshold 0.5 is set, new matches can be declared. Since these new matches are obtained with the threshold increases, the landmarks represented by these new matches are assigned a weight slightly less than 1. Repeated steps are performed until the maximum threshold 0.8 is set, and then the weighted SIFT matches can be generated. The parameter settings we select for multi-level matching are shown in Table 1, where τ represents the weight for multi-level matching.

Similar to Strategy 2, multi-level matching is also a strategy for weight evaluation, which is appropriate and effective for visual homing. Under the premise that the total number of landmarks is limited, multi-level matching further highlights the value of the high-quality landmarks.

Based on Strategies 2 and 3, the optimized landmarks will carry two types of weight information, namely *w* and *τ*. Therefore, for the *i*th landmark ***v****_i_*, the landmark vector it forms is no longer the traditional unit vector, the modulus of the vector becomes the product of *w_i_* and *τ_i_*:

(12) $$\left|v_{i}\right|=w_{i}\cdot \tau_{i}\;$$

After the moduli of all landmark vectors are calculated, the optimal ALV computed in the proposed method becomes in fact a weighted average of landmark vectors, and the final homing vector can be similarly generated based on Equation (5).

### 3.4. Overview of the Proposed Strategies

**Figure 6:**
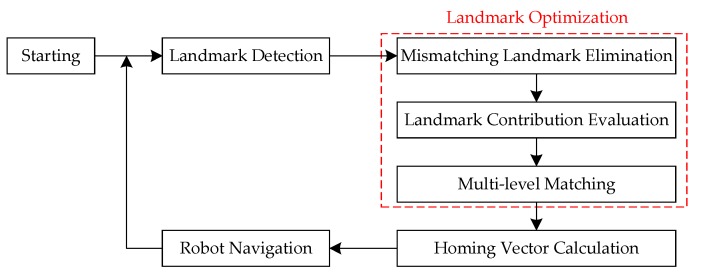
Procedure of the mobile robot’s visual homing process based on the proposed strategies.

Figure 6 shows the complete procedure of the mobile robot’s visual homing process based on the proposed strategies. The details are characterized as follows:

- Landmark Detection: SIFT features are detected and matched from the currently captured image and pre-stored target image, all SIFT matches are considered as the original landmarks.
- Landmark Optimization: Based on the proposed three strategies, the original landmarks are optimized in terms of precision and distribution.
- Homing Vector Calculation: A certain visual homing approach is adopted to calculate the homing vector based on the optimized landmarks. It should be noted that, although we only use ALV in this paper, the proposed strategies can also apply to other landmark-based visual homing approaches.
- Robot Navigation: The mobile robot is guided by the calculated homing vector to move a preset step, and then re-performs the process of Landmark Detection until the target location is reached.

## 4. Experiments

### 4.1. Experimental Environment and Mobile Robot Platform

**Figure 7:**
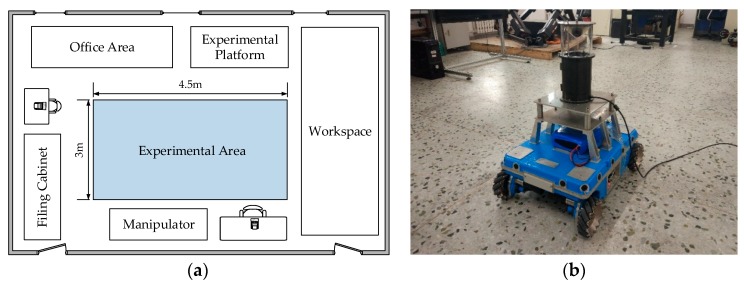
Experimental environment and mobile robot platform: (**a**) experimental environment, where the grey part denotes the experimental area; and (**b**) mobile robot platform.

To verify the homing performance of the proposed strategies, related homing experiments were carried out. The experimental environment is shown in Figure 7a. The experimental area of the robot is a 4.5 m × 3 m rectangular space, surrounded by different types of objects (such as tables, filing cabinets and other experimental equipment). The mobile robot platform is shown in Figure 7b. A panoramic vision system is installed on the omni-directional mobile robot. The robot is also equipped with four mecanum wheels, which can help the robot move towards any direction without changing orientation.

**Figure 8:**
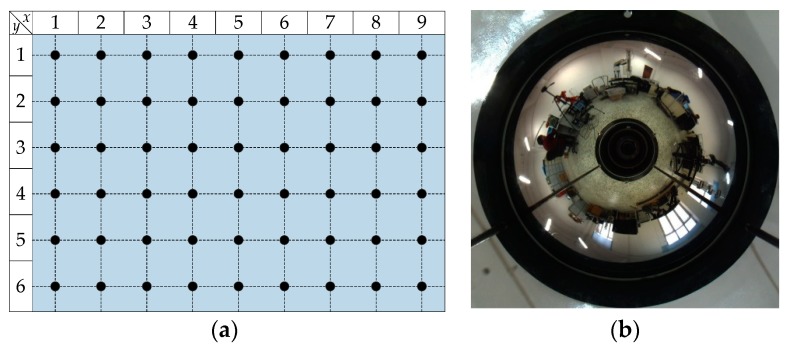
Representative locations and panoramic image sample: (**a**) representative locations, which are denoted as the black solid circles; and (**b**) panoramic image sample, which was captured at Location (8,4).

A set of image databases was collected based on the default experimental environment. We selected 9 × 6 = 54 representative locations in the experimental area, which is shown in Figure 8a. The distance between two adjacent locations was set to 50 cm, and all the panoramic images were collected. During the image collection, the environmental conditions should always be unmodified, and the robot’s orientation was artificially controlled to remain constant. We used surveying tools to mark the 54 representative locations in advance, and then placed the robot on each marked location to capture corresponding image. To keep the robot’s orientation as constant as possible, the stitching lines between floor tiles can be regarded as a reference, and a reliable external compass can also be applied. Figure 8b shows a panoramic image sample, the resolution of all the images is 768 × 768.

### 4.2. Performance Indicators

Four widely used performance indicators are adopted in this paper: *AAE* (average angular error), *RR* (return ratio), *TDE* (travel distance error) and *ATS* (average trajectory smoothness).

*AAE* is adopted to measure the average difference between the calculated homing direction *α* and the desired homing direction *β* (i.e., the direction pointing from *C* to *T*). Taking *C* as the origin of the world coordinate system, *α* and *β* can thus be defined as follows:

(13) $$\alpha =\mathrm{atan}\;2\;(h_{y},h_{x})\;$$

(14) $$\beta =\mathrm{atan}\;2\;(y_{T}-y_{C},x_{T}-x_{C})\;$$

where (*h_x_*,*h_y_*) is the coordinate of the calculated homing vector. (*x_C_*,*y_C_*) and (*x_T_*,*y_T_*). respectively. denote the coordinates of *C* and *T*. Therefore, the angular error *AE*(*T*,*C*) can be calculated as follows:

(15) AE (T,C)=|α−β| 

where we stipulate *AE*(*T*,*T*) = 0. To obtain a more general result, researchers often select multiple possible current locations to test the overall homing performance of a certain visual homing approach. Assuming that the total number of the selected current locations is *q*, so the average angular error *AAE*(*T*,*C*) can be calculated by:

(16) $$AAE\;(T,C)=\frac{1}{q}\sum_{i=1}^{q}AE\;(T,C_{i})\;$$

where *C_i_* is denoted as the *i*th current location. Obviously, low *AAE* value indicates that the robot can move towards the target location with the trajectory closer to a straight line.

*RR* is a performance indicator measuring the homing success rate of the mobile robot. For a certain starting location, a dummy robot is created to perform a simulated movement based on the calculated homing vector, and if the robot can continually move a pre-set step until the target location is reached, we declare the homing process successful. However, if the robot moves more than half the perimeter of the experimental area or outside the boundary, we declare the homing process failed. For all the *t* possible current locations, the *RR* value can be calculated by:

(17) $$RR=\frac{t\prime }{t}\;$$

where *t*′ represents the total number of times that the target location can be successfully reached. Obviously, high *RR* value indicates that the robot can return to the destination from more locations.

*TDE* is adopted to measure the difference between the actual travel distance of the mobile robot and the ideal situation. If a certain homing process is declared successful, the total travel distance of the mobile robot *d_act_* is measured and the ideal minimum distance *d_ide_* is calculated, then the *TDE* value can be easily generated by:

(18) $$TDE=\left|d_{act}-d_{ide}\right|\;$$

*TDE* is applicable to the actual homing experiments, and the deviation of the robot’s motion can be evaluated by measuring the actual trajectory. Low *TDE* value indicates that the robot can reach the target location with high efficiency.

**Figure 9:**
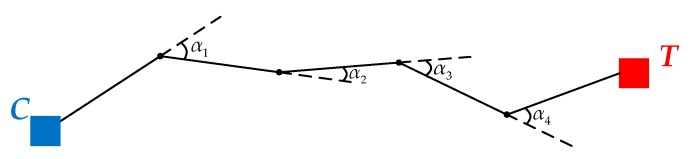
A certain actual trajectory of mobile robot. The black solid line represents the actual trajectory. The black dots represent the location where the robot recalculates the homing vector. *α*_1_, *α*_2_, *α*_3_ and *α*_4_ denote the offset angles.

*ATS* is adopted to evaluate the smoothness of the generated trajectory. Assuming that a certain actual trajectory of mobile robot is shown in Figure 9, and the offset angle can be defined as the complementary value of the angle between two adjacent segments.

Assuming that the total number of the robot’s movement is *N*, it can thus be indicated that *N* − 1 offset angles can be obtained, denoted as *α*_1_, *α*_2_, …, *α_N_*_−1_. Therefore, the *ATS* value can be computed by:

(19) $$ATS=\frac{1}{N-1}\sum_{i=1}^{N-1}\alpha_{i}\;$$

Same as *TDE*, *ATS* is also a performance indicator applicable to the actual homing experiments, and the smoothness of the trajectory can be calculated based on Equation (19). Low *ATS* value indicates that the robot can reach the target location more robustly.

### 4.3. Homing Experiments on Image Databases

**Figure 10:**
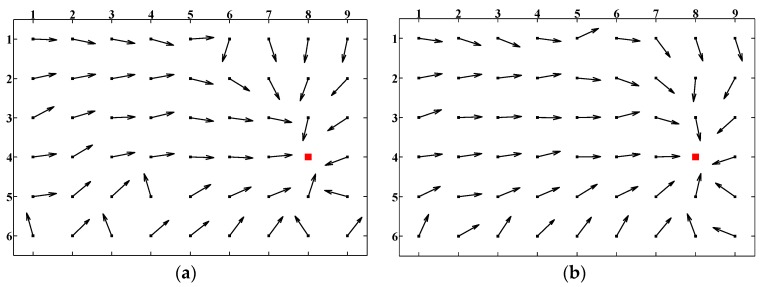

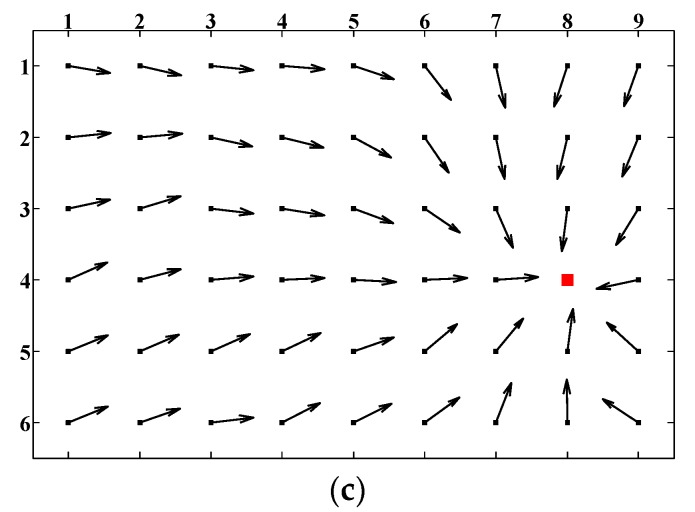
Homing vector fields (50 cm spacing): (**a**) homing vector field of ALV; (**b**) homing vector field of SL-ALV; and (**c**) homing vector field of the proposed strategies. Red squares denote the pre-set target location. Black arrows represent the calculated homing vectors pointing from the possible current location to the target location.

Homing experiments were performed based on the image databases introduced in Section 4.1. To evaluate the overall homing performance, we arbitrarily selected a representative location as the target location while the remaining 53 locations were considered as all possible starting locations. By employing the visual homing approaches, the homing vectors were generated from each possible starting location to the target location, and we call this kind of visualization results as the homing vector field. Figure 10 shows the three homing vectors fields of ALV, SL-ALV and the proposed strategies based on image databases. Location (8,4) was selected as the target location. It can be seen intuitively from the figure that both SL-ALV and our proposed strategies can improve the homing performance of the original ALV algorithm, but obviously the homing vector field of our proposed strategies is neater, as the number of arrows that can accurately point to the target location is relatively more, thus it can be inferred that the calculated homing vector is closer to the ideal situation. Therefore, our proposed strategies outperform both ALV and SL-ALV.

**Figure 11:**
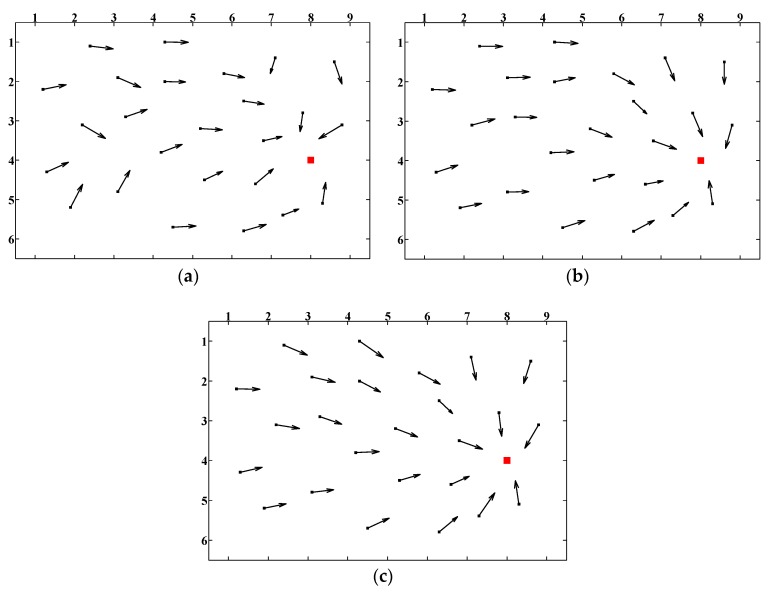
Homing vector fields (random spacing): (**a**) homing vector field of ALV; (**b**) homing vector field of SL-ALV; and (**c**) homing vector field of the proposed strategies.

We also performed experiments when the spacing between two adjacent current locations is random. We arbitrarily selected 25 locations as the possible current locations, and the principle of the image collection remained unchanged. Figure 11 shows the three homing vector fields of ALV, SL-ALV and the proposed strategies when the spacing is random. The conclusions that can be drawn are the same as those in Figure 10: our proposed strategies can still exhibit better homing performance than the ALV and SL-ALV algorithm.

**Figure 12:**
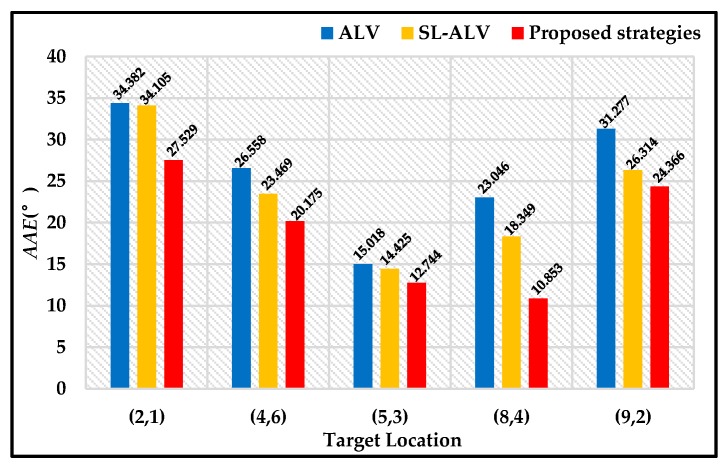
*AAE* results for ALV, SL-ALV and proposed strategies. The blue histograms denote the original ALV algorithm. The original histograms denote SL-ALV. The red histograms denote the proposed strategies.

To evaluate the *AAE* results, we selected Location (2, 2), (4, 6), (5, 3) and (7, 1) as another four different target locations. We selected the above locations as different target locations because these five locations were distributed almost evenly in the experimental area, making the conclusions more convincing. The *AAE* results of the above five locations are shown in Figure 12. Compared to the original ALV algorithm, our proposed strategies can reduce the *AAE* value by 26.57% in total, while SL-ALV can only reduce the *AAE* value by 10.45%. Therefore, it can be concluded that the proposed strategies have better optimization effects than SL-ALV.

**Figure 13:**
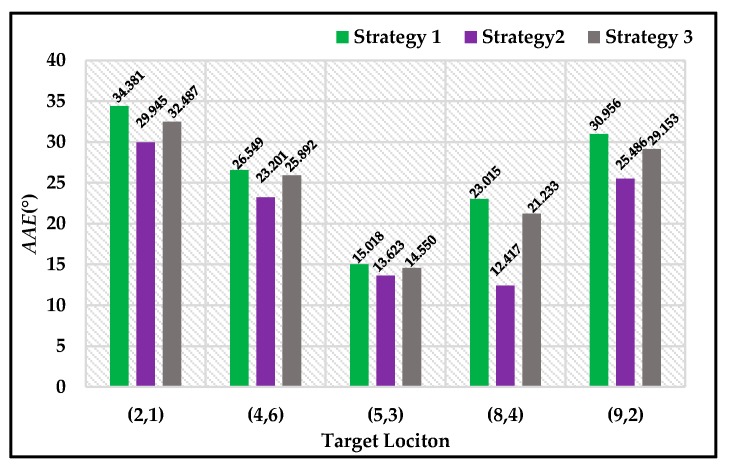
*AAE* results when the three proposed strategies were applied separately. The green histograms denote Strategy 1, the purple histograms denote Strategy 2, and the grey histograms denote Strategy 3.

In addition, we also evaluated the *AAE* results when the three proposed strategies were applied separately, as shown in Figure 13. It can be seen that all the three strategies can optimize the homing performance. Among them, the optimization effect of Strategy 2 is the most obvious, and Strategies 1 and 3 can improve the homing performance slightly.

To evaluate the *RR* results, we considered each location as a possible target location, and repeatedly calculated 54 sets of *RR* values. The statistics of *RR* values for all the 54 possible target locations are shown in Table 2, and the conclusions that can be drawn are similar to those in Figure 10, Figure 11, Figure 12 and Figure 13. Compared to SL-ALV, our proposed strategies can further improve the *RR* values, so the robot can move from more possible locations to the target location.

We also tested the average time required for the above three algorithms to calculate a homing vector (Inter Core i7-6800K 3.4 GHz, MATLAB R2012a), as shown in Table 3. It can be seen that these three algorithms have almost the same calculation speed; the proposed strategies do not significantly increase the calculation amount.

### 4.4. Homing Experiments on Mobile Robot

To further compare the homing performance of SL-ALV and our proposed strategies, we performed a series of homing experiments on a real mobile robot. Since the robot’s orientation might be slightly offset in actual experiments, it would be meaningful to evaluate the homing performance in practical applications. We arbitrarily selected three discrete locations as the target locations, and for each target location, three different starting locations were also selected. The robot was placed at the target location in advance to capture the current panoramic image. During the experiment, the robot’s orientation remained constant, and the environment remained stationary. After the preparation was completed, the robot was placed at the starting location to be tested.

The detailed experimental steps are as follows:

*Step* *1:* The robot captures and stores the currently viewed panoramic image.
*Step* *2:* Two stored panoramic images are matched by the SIFT algorithm, and the homing direction are obtained by SL-ALV/ our proposed strategies.
*Step* *3:* The robot travels 30 cm along a straight line, and then pauses.
*Step* *4:* If either of the following cases happens, jump to *Step 6*.
  *Case* *1:* The robot arrives at a range of 30 cm centered on the target location.
  *Case* *2:* The robot moves more than 750 cm or out of the experimental area.
*Step* *5:* Jump to *Step 1*.
*Step* *6:* If *Case 1* happens, we declare the homing process successful. If *Case 2* happens, we declare the homing process failed.
*Step* *7:* The robot is manually stopped.

**Figure 14:**
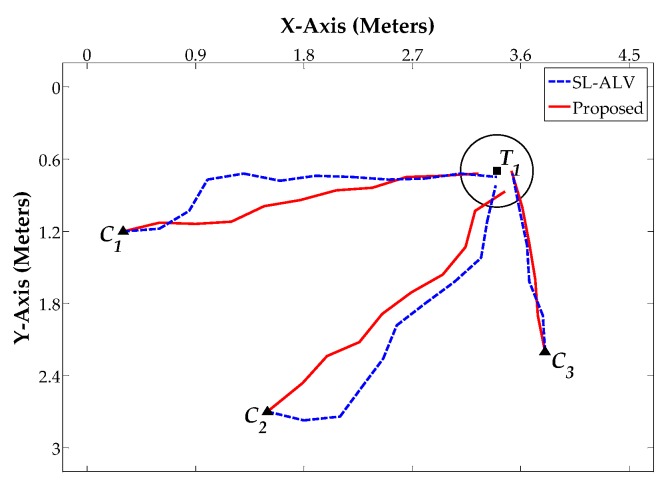
Homing Experiment 1. The target area is denoted as a circle centered on a square block. The three starting location *C*_1_, *C*_2_ and *C_3_* are marked as triangular blocks. Blue dashed lines denote the homing trajectories generated by SL-ALV. Red solid lines denote the homing trajectories generated by our proposed strategies.

**Figure 15:**
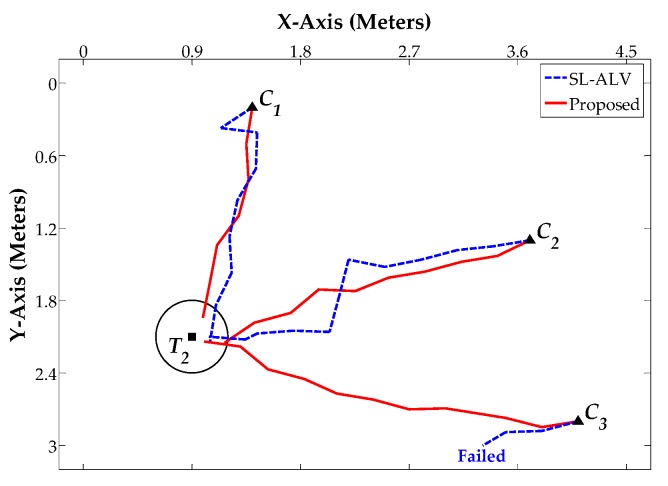
Homing Experiment 2.

**Figure 16:**
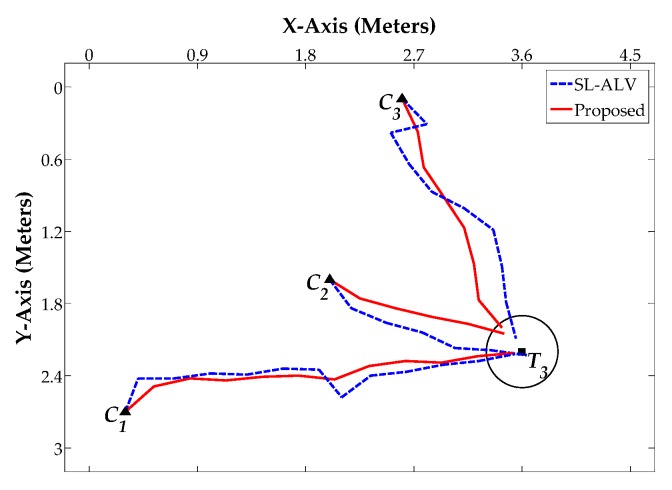
Homing Experiment 3.

Figure 14, Figure 15 and Figure 16 show the robot’s trajectories by SL-ALV and our proposed strategies, and Table 4, Table 5 and Table 6 show the corresponding results recording the *AAE*, *ATS*, *TDE* values and the total number of the robot’s movement steps *N*. As can be seen intuitively from the figures and tables, the trajectories generated by our proposed strategies are smoother than those generated by SL-ALV, the robot can reach the target location with the trajectory closer to a straight line. In total, the homing processes by our proposed strategies are all successful, while SL-ALV has a homing failure. For the eight sets of homing experiments where both SL-ALV and our proposed strategies have successfully controlled the robot to the target area, the experimental results can be summarized as follows: First, the average *AAE* of SL-ALV is 17.02°, while this value of our proposed strategies is only 9.31°, the angular error can be reduced by about 45.30%. Second, the average *ATS* of SL-ALV is 25.64°, while this value of our proposed strategies is only 17.52°, the offset angle can be reduced by about 31.67%. Third, the total number of homing steps for SL-ALV is 72, while, for our proposed strategies, this number is only 62. Fourth, the total *TDE* value of our proposed strategies is 2.29 m, while, for our proposed strategies, this number is only 0.35 m. Overall, the homing experiments on a real mobile robot indicate that the homing effects by our proposed strategies are more accurate, and the robot’s movement processes are smoother.

## 5. Discussion

In Section 4, we performed the related experiments to evaluate the homing performance of ALV, SL-ALV and our proposed strategies. The experiments consist of two aspects, one based on the image databases and the other based on the real mobile robot. For the experiments on image databases, the homing performance of the algorithms was evaluated under ideal conditions, the experimental environment was always static and the robot’s orientation was almost guaranteed to be constant. For the experiments on mobile robot, the homing performance of the algorithms was evaluated under actual conditions, the robot’s orientation might be slightly offset during the movement. According to the experimental results, our proposed strategies were verified to exhibit good performance both in theory and in practice. The simulation results of our proposed strategies had low *AAE* values and high *RR* values, while the robot’s trajectories generated by our proposed strategies had low *TDE* values and low *ATS* values.

However, in the process of the experiments, we also found two problems that cannot be solved at present. On the one hand, the environment should always be static. If dynamic situations occurred (for example, during the movement of the robot, the researchers were randomly moving and the positions of some objects were arbitrarily changed), the optimization effect would be reduced. On the other hand, the mobile robot should always be subject to holonomic constraints. The performance of all the mentioned homing algorithms would significantly drop under nonholonomic constraints, such as randomly changing the robot’s orientation and vertical height.

For the emergence of the above problems, we believe that it is related to the basic concept of visual homing. Since traditional visual homing relies on only a panoramic vision sensor to complete the navigation task, the application scope of this technique will be relatively limited. Although our proposed strategies can effectively improve the homing performance, it is an algorithm-level optimization after all. As far as we know, most existing visual homing approaches require control variables, such as static environments, illumination changes, holonomic constraints, etc. Some papers have proposed improvements to solve these problems, and visual homing can now cope with the problem of illumination changes well by adopting scale-invariant features. However, in terms of dealing with dynamic environments and nonholonomic constraints, although relevant solutions have made progress, some defects still exist, such as poor performance and excessive calculation [17,28,41]. Therefore, it will be valuable to further improve the performance of visual homing in these aspects.

## 6. Conclusions

In this paper, we propose three landmark optimization strategies for mobile robot visual homing. Two outstanding advantages of our strategies can be summarized. On the one hand, to avoid potential problems that may arise due to the excessive landmark elimination, our strategies adopt the method of weight assignment, and the landmark distribution can thus be optimized by adjusting the contribution of each landmark. On the other hand, our strategies have almost no influence on the calculation speed, and the robot can still reach the target location with high efficiency.

Homing experiments were carried out on both image databases and a real mobile robot. The results reveal that our proposed strategies can better improve the homing performance of ALV. By using our proposed strategies, the *AAE* values can be further reduced and the *RR* values can be further increased. In practical applications, the robot can reach the target location with a more ideal trajectory, and the *TDE* and *ATS* values can also be further reduced.

In the future, we will conduct research from the following three aspects: First, we will be more concerned with improving the effect of the visual homing methods in more complex (such as the presence of obstacles or dynamic objects) or larger environments. Second, instead of artificially setting too many key parameters (such as Strategy 3), we will explore more intelligent solutions to adaptively determine parameters by sensing environments. Third, we will consider the homing performance for more general mobile robots, and solve the problem that the effect will decrease under nonholonomic constraints.

## Figures and Tables

**Table 1:** Parameter settings for multi-level matching.

**Interval**	[0,0.4)	[0.4,0.5)	[0.5,0.6)	[0.6,0.7)	[0.7,0.8]
**τ**	1.00	0.95	0.90	0.85	0.80

**Table 2:** Statistics of *RR* values.

Algorithm	Min	Max	Mean	Quartile		
				*Q*_1_	*Q*_2_	*Q*_3_
ALV	0.389	1.000	0.812	0.736	0.868	0.906
SL-ALV	0.415	1.000	0.845	0.792	0.868	0.962
Proposed strategies	0.433	1.000	0.877	0.792	0.962	1.000

**Table 3:** Average time required to calculate a homing vector.

Algorithm	Time (s)
ALV	1.124
SL-ALV	1.083
Proposed strategies	1.275

**Table 4:** Associated results of homing experiment 1.

Location	Algorithm	Success	*AAE* (°)	*ATS* (°)	*N*	*TDE* (m)
*C*_1_	SL-ALV	Yes	11.59	18.26	11	0.17
	Proposed	Yes	5.94	11.92	10	0.03
*C*_2_	SL-ALV	Yes	20.03	20.79	10	0.35
	Proposed	Yes	13.23	17.86	9	0.08
*C_3_*	SL-ALV	Yes	11.52	10.69	5	0.01
	Proposed	Yes	11.39	5.10	5	0.01

**Table 5:** Associated results of homing experiment 2.

Location	Algorithm	Success	*AAE* (°)	*ATS* (°)	*N*	*TDE* (m)
*C*_1_	SL-ALV	Yes	27.67	47.31	8	0.45
	Proposed	Yes	9.37	15.90	6	0.04
*C*_2_	SL-ALV	Yes	17.86	26.14	11	0.38
	Proposed	Yes	9.54	18.49	9	0.06
*C_3_*	SL-ALV	No	——	——	——	——
	Proposed	Yes	8.43	14.55	11	0.07

**Table 6:** Associated results of homing experiment 3.

Location	Algorithm	Success	*AAE* (°)	*ATS* (°)	*N*	*TDE* (m)
*C*_1_	SL-ALV	Yes	14.28	26.56	12	0.07
	Proposed	Yes	7.97	13.02	11	0.01
*C*_2_	SL-ALV	Yes	10.78	14.93	6	0.34
	Proposed	Yes	8.29	6.23	5	0.08
*C_3_*	SL-ALV	Yes	22.40	40.45	9	0.52
	Proposed	Yes	8.73	16.60	7	0.04
